# The Effects of Essential Oils and Terpenes in Relation to Their Routes of Intake and Application

**DOI:** 10.3390/ijms21051558

**Published:** 2020-02-25

**Authors:** Sachiko Koyama, Thomas Heinbockel

**Affiliations:** 1 Department of Biology, Indiana University, Bloomington, IN 47405, USA; 2 Department of Anatomy, College of Medicine, Howard University, Washington, DC 20059, USA

**Keywords:** olfactory system, skin, gastro-intestine system, essential oils, oil constituents, terpenes, neuromodulation

## Abstract

Essential oils have been used in multiple ways, i.e., inhaling, topically applying on the skin, and drinking. Thus, there are three major routes of intake or application involved: the olfactory system, the skin, and the gastro-intestinal system. Understanding these routes is important for clarifying the mechanisms of action of essential oils. Here we summarize the three systems involved, and the effects of essential oils and their constituents at the cellular and systems level. Many factors affect the rate of uptake of each chemical constituent included in essential oils. It is important to determine how much of each constituent is included in an essential oil and to use single chemical compounds to precisely test their effects. Studies have shown synergistic influences of the constituents, which affect the mechanisms of action of the essential oil constituents. For the skin and digestive system, the chemical components of essential oils can directly activate gamma aminobutyric acid (GABA) receptors and transient receptor potential channels (TRP) channels, whereas in the olfactory system, chemical components activate olfactory receptors. Here, GABA receptors and TRP channels could play a role, mostly when the signals are transferred to the olfactory bulb and the brain.

## 1. Aromatherapy and Essential oils

Scientific studies on the chemical constituents of essential oils have just started in the 20th century, although the history of using essential oils as medical agents or for relaxation goes back to ancient times in Egypt. It was only early in the 20th century that the word “aromatherapy” was coined by the French chemist René-Maurice Gattefossé for treatments using essential oils. He was a chemist working at his family-owned perfume company in France, and not a clinician of holistic, alternative medicine. An anecdotal story tells that he had accidentally burnt his hand because of an explosion in the laboratory and happened to use an essential oil of lavender, which suppressed the pain and scar formation. This experience led him to study the possibility of using essential oils for therapeutic purposes. His book “Gattefossé’s Aromatherapy” [1] is perhaps the earliest book published on aromatherapy by a scientist and it contains a substantial amount of description on terpenes.

“Essential oils contain constituents which possess almost the full range of chemical functions. The simplest are hydrocarbons, constituting the terpene family, of the type *p*-cymene, C_10_H_14_, which is similar to pinene, a constituent of oil of turpentine. The other constituents can almost all be classified as various stages in terpene development” [1].

It has been eighty years since then, but we still need thorough scientific studies on the effect of essential oils, i.e., their effects on healing diseases, or delaying the progress of disease, and on improving mental conditions, as they are well known to do. We can now examine their effects with modern techniques and advanced knowledge. We need studies on the chemical compounds involved in producing these effects and on the mechanisms of action, as well as studies on the routes involved.

As defined, “aromatherapy” is “the use of essential oils from plants (flowers, herbs, or trees) as therapy to improve physical, mental, and spiritual well-being” (from NIH, National Cancer Institute website; https://www.cancer.gov/about-cancer/treatment/cam/patient/aromatherapy-pdq#_1). Essential oils are used (1) by inhaling, which will use mostly the olfactory system, and to some extent the skin, as the chemical compounds will reach the skin. (2) By topically applying essential oil on the skin, in which the major route will be through the skin, and to a lesser extent through the olfactory system, as the aroma will reach the olfactory system. (3) By drinking, in which the major route will be through the digestive system and, secondarily, because of the retro-nasal location of the nose, through the olfactory sensory system, as the aroma will reach the nose from the mouth and stomach. As such, when essential oils are used, there are multiple routes involved. The routes involved in generating a certain effect depend on the types of chemical compounds included in the essential oil. For example, recently, topical application of β-caryophyllene, a sesquiterpene, in various herbs and spices, was found to improve re-epithelialization of cutaneous wounds, whereas exposure solely through the air did not produce that impact [2]. Linalool (3,7-dimethylocta-1,6-dien-3-ol), a monoterpene found in herbs, spices, and fruits, has an anxiolytic effect, and this effect is mediated through the olfactory system [3]. β-caryophyllene and linalool are both present in, for example, lavender essential oils. These examples, and the fact that they are both included in the same essential oil, indicate that it is important to know what effect each chemical compound has, and which route is involved in producing these effects. This also indicates that the method of using essential oils should be adjusted depending on the chemical compounds that are expected to function: for example, it is critical to use a diffuser for lavender essential oil when the expected effect is the anxiolytic impact of linalool. In contrast, lavender essential oil should be applied topically when the goal is to enhance wound healing by β-caryophyllene. It is necessary to use the delivery method that is most effective to reach the clinical goals with the knowledge on how specific chemical compounds produce the effect. Therefore, it is pivotal to determine the types of impacts that each chemical compound generates, what routes are involved in these impacts, and the mechanisms of action.

Considering that the olfactory system, the skin, and the digestive system are the three possible routes through which essential oils can affect physiological and psychological health conditions, we will summarize the olfactory system, the skin, and the digestive system, respectively. Olfactory receptors are expressed in non-olfactory tissues as well, including the skin and the gastro-intestinal system, both located outside of the olfactory system [4]. In addition, there are non-olfactory receptors in the skin and the gastro-intestinal system that are activated by odorous chemical compounds [5]. We will discuss these respectively.

An essential oil is a “product obtained from a natural raw material of plant origin by steam distillation, by mechanical processes from the pericarp of citrus fruits, or by dry distillation” (definition by International Organization for Standardization (ISO); 9235:2013). Terpenes are the largest group of components in essential oils [6]. Depending on the number of isoprene units (C_5_H_8_) in the molecule, they are classified into, for example, monoterpenes (one terpene unit or two isoprene units; examples are linalool, geraniol, limonene), sesquiterpenes (three isoprene units; examples are β-caryophyllene, the farnesenes, humelene), and tetraterpenes (eight isoprene units; for example, carotenoids). Monoterpenes and sesquiterpenes comprise about 25% of the terpene fractions in essential oils [6]. We will summarize the essential oils, the terpenes found in essential oils, and their impacts on physiological or psychological status.

## 2. Olfactory System

Studies on the olfactory system have shown a rapid increase in numbers during the past 30 years. We now know how we can distinguish the large number of different types of odorants surrounding us in the environment (over 10^12^ odorant chemical compounds) [7,8]. The morphology of the olfactory epithelium as well as the olfactory bulb and the routes of signaling from the sensory neurons to the brain have been uncovered. Studies have found that odors (odorants and pheromones) can affect the behaviors and physiological conditions of the receiver of the odors, and the mechanisms of action of some of these phenomena are starting to be determined. We now know that the neuroendocrine and endocrine systems are involved in regulating olfactory learning and the olfactory sense.

**Figure 1:**
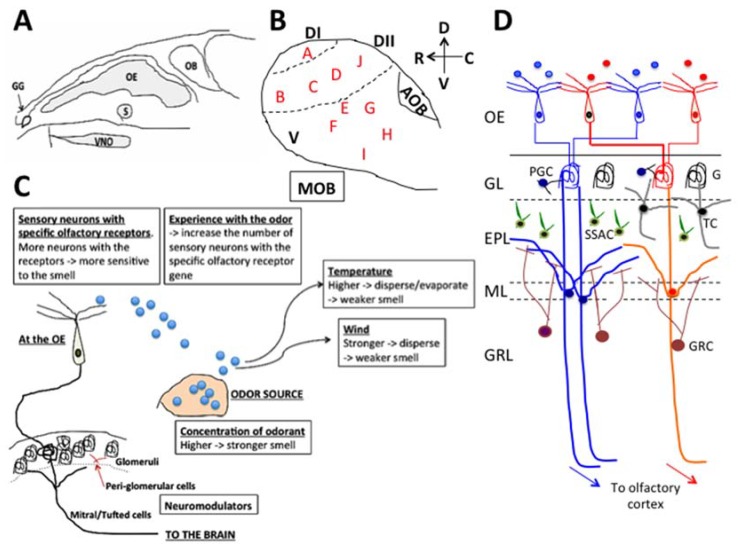
The olfactory system. Locations of areas with sensory neurons in the nasal cavity of mice (**A**), the domains of the olfactory bulb with clusters of odor maps (**B**), a summary of the factors that affect the olfactory sense (**C**), and the pathway in the olfactory bulb (**D**). OB: olfactory bulb, OE: olfactory epithelium, GG: Grüneberg ganglion, S: septal organ, VNO: vomeronasal organ, GL: granular layer, EPL: external plexiform layer, ML: mitral cell layer, GRL: granule cell layer, PGC: peri-glomerular cell, G: glomerulus, TC: tufted cell, GRC: granule cell, SSAC: superficial short axon cell. The letters in red in (**B**) indicate the locations of Cluster A to J based on Mori et al. [28] and Mori and Sakano [29]. The D, V, R, C on the upper right side top in (B) beside the arrows mean dorsal, ventral, rostral, caudal, respectively.

In contrast to the limited number of receptor types that sense light for vision, there are over 1000 different types of functional olfactory receptor genes in the case of mice [9,10] and less than 400 types in the case of humans [10,11,12,13]. The olfactory receptor proteins are expressed in the cilia of olfactory sensory neurons and each olfactory sensory neuron in the nose expresses a single type of olfactory receptor gene, which contributes to the main role of the olfactory system, namely, to detect and distinguish odorants. In the case of mice, the sensory neurons reside in multiple regions in the nasal cavity (Figure 1A), i.e., the main olfactory epithelium, vomeronasal organ, septal organ of Masera, and Grüneberg ganglion [14,15]. In addition, there are separate sensory receptors expressed in other sensory neurons that reside in the main olfactory epithelium or vomeronasal organ, but carry different receptor types. These are the receptor guanylyl cyclase (GC-D) [16] and trace amine-associated receptors (TAAR) [17,18] in the main olfactory epithelium and formyl peptide receptors in the vomeronasal organ [18,19]. The sensory neurons project their axons to the olfactory bulb. The projection targets in the olfactory bulb are specific for the locations where the sensory neurons originated in the olfactory epithelium [20,21], and for the types of olfactory receptors (e.g., the GC-D and TAAR). GC-D and TAAR sensory neurons project their axons to separate locations in the olfactory bulb compared to where the axons of olfactory neurons project to, although they share the same olfactory epithelium [16,17,18]. The area in the olfactory bulb, where axons of olfactory sensory neurons in the main olfactory epithelium terminate, is called the main olfactory bulb (MOB), whereas the area where vomeronasal neurons project to is called the accessory olfactory bulb (AOB). Other than these two major areas, there are necklace glomeruli (NG). NGs are the glomerular axonal targets of sensory neurons from the Grüneberg ganglion (G) [22,23], TAAR neurons, and GC-D neurons that generate glomeruli [24] around the AOB in the shape of a necklace (hence the name ‘necklace’ glomeruli). In the olfactory bulb, the axon terminals construct glomeruli and synaptically connect to mitral cells or tufted cells [16,20,24,25]. The main role of the olfactory bulb is to organize the information detected at the sensory neuron level located in the multiple olfactory systems in the nasal cavity and to process the information in the first relay for olfactory information in the brain. The path of the axons of olfactory sensory neurons in the main olfactory epithelium to the MOB is classified into four different zones, Zone 1 to Zone 4 from the dorsal region to the ventral region of the MOB [20,26]. The locations of the olfactory sensory neurons, which project their axons, are also distributed in zone-specific ways in the main olfactory epithelium, from the dorsal area to lateral/ventral areas [20,26]. The axons from the vomeronasal neurons also show zone specificity, i.e., those running from the apical region of the vomeronasal organ project to the rostral domain in the AOB, whereas those from the basal region project to the caudal domain in the AOB. At the end of 20th century, a new neural cell adhesion molecule, known as the olfactory cell adhesion molecule (OCAM), was found. The expression of OCAM showed zone differences, i.e., negative in Zone 1 but positive in Zones 2 to 4 in the main olfactory epithelium and in the MOB, and positive in the apical region of the vomeronasal organ and rostral region of the AOB, but negative in the basal region of the vomeronasal organ and caudal AOB [26]. Based on these findings, the names of Zone 1 to 4 of the olfactory epithelium are often called the Dorsal domain (Zone 1; OCAM negative) and the Ventral domain (Zones 2 to 4; OCAM positive). In addition, some olfactory receptors are expressed across Zones 2 to 4, which also supports the new classification of the Dorsal domain and the Ventral domain ([27] and personal communication with Dr. Yoshihara of Riken, Japan). In the Dorsal domain, areas are targeted by axons of TAAR neurons and axons of Class I olfactory receptors, which further separates the Dorsal domain into Dorsal domain I (DI) and Dorsal domain II (DII) ([28] and personal communication of Dr. Yoshihara of Riken, Japan) [28] (Figure 1B black color DI and DII).

Some hypotheses suggest that functional differences exist depending on these domain differences [29]. Studies show that domain-dependent differences exist in the odorants that activate glomeruli [30,31]. The odors that activate the glomeruli in each of these domains are becoming clearer [30,31] and are classified into Clusters A to I in the olfactory bulb [30] (Figure 1B red color A to I). The DI domain is where odor Cluster A is located, and the glomeruli there respond to chemical compounds of amines and fatty acids. DII is located between DI and the ventral domain, and odor Clusters B, C, D, and J are included in DII [31]. Odor Clusters B, C, D, and J are the clusters that house glomerular neurons that respond to aliphatic alcohols (Cluster B), phenol family odorants (Cluster C), and a variety of ketones (Cluster D). It should be noted that neurons in glomeruli in Cluster J respond to predator odors, such as trimethyl-thiazoline (TMT), and neurons in glomeruli in Cluster D and Cluster J respond to various pheromones (for example, 2-sec-tutyl-dihydrothiazole (SBT) and dehydro-exo-brevicomine (DHB) and other male urine odorants) [31]. Mori and Sakano [31] found that the odors detected in the ventral domain, which includes odor Clusters E, F, G, H, and I, are methoxy pyrazines, green odorants, C6 and C9 compounds, isothiocyanates, **terpene hydrocarbons**, esters, **terpene alcohols**, and sulfides (foods, fruits, and vegetables). This suggests that the area where terpenes in essential oils are sensed in the olfactory epithelium could be the lateral/ventral areas, which project their axons from sensory neurons to the lateral/ventral domain in the olfactory bulb. This hypothesis is partially supported using three types of terpene compounds, cavone, 1,8-cineole, and limonene, and the different responses generated that depend on the types of compounds. The 1,8-Cineole and limonene generated responses in the lateral/ventral part of the olfactory epithelium whereas the responses to cavone included the dorsal region and the lateral/ventral region [32]. Table 1 summarizes the types of odorous chemical compounds that activate glomeruli in each cluster. The locations of the clusters are shown in Figure 1B.

In relation to zones in the olfactory epithelium and domains in the olfactory bulb, it should be noted that certain odors activate glomeruli in separate domains in the olfactory bulb, some glomeruli in the dorsal domain, and some glomeruli in the ventral domain [31]. Their hypothesis on these multiple projections is that the dorsal domain is involved in fear responses, in contrast to the lateral/ventral domain, which is involved in learning, thus, related to the multiplicity in the roles of the routes [31]. Another point to be noted is that, although most olfactory receptors become activated by small sub-groups of chemical compounds with similar chemical structure, some olfactory receptors are activated by a rather broad range of structurally unrelated chemical compounds. The odorants that activate Olfr124 (also known as SR1 and MOR256-3) include, for example, hexanoic acid (caproic acid), octanal, valeric acid, (+) camphor, fox urine, (+) limonene, lyral (trade name of the synthetic aroma compound, 4-(4-Hydroxy-4-methylpentyl)cyclohex-3-enecarbaldehyde), and β-pinene [33]. These studies suggest that the multiplicity of the roles of the olfactory system can be generated both at the level of the olfactory epithelium and of the olfactory bulb. The function of this multiplicity could be related to mood (fear) vs. learning [29,31], homeostatic condition [34], and conspecific olfactory communication [35]. Region dependent analyses of neuromodulators in the olfactory bulb may provide important information that increases understanding of the functions of each zone/domain in the olfactory bulb, and may provide information on the mechanisms of action of the essential oil constituents.

Sensitivity to odors depends on features related to the nature of the odors and the environment. For example, the features on the odor side include the concentration of the odor, hydrophilic/hydrophobic nature of the chemical compound, the humidity, which affects evaporation rate, and the air flow (winds), which affects how much of the odor can reach receptors. The features on the sensory neuron side are, for example, the number of olfactory neurons that carry the specific olfactory receptor for the detection of a specific type of odor. The features that produce individual differences are, for example, previous experiences with the odor in past generations [36,37] and during the individual’s life [38,39]. Physiological conditions, such as estrous status, also affect the olfactory sense, indicating that neuromodulators or hormones affect olfactory ability and acuity [40,41] (Figure 1C). These features and factors need to be controlled or taken into consideration when studying the effects of aromatic chemicals.

With reference to signaling pathways, it has been known from early in the 1970s that the olfactory bulb is histologically separated in multiple layers [42], which are, from the outside, the glomerular layer, the external plexiform layer, the mitral cell layer, the internal plexiform layer, and the granule cell layer (Figure 1D). The axons that project to the olfactory bulb from the sensory neurons in the nasal cavity construct glomeruli and synaptically connect to mitral/tufted cells, which, in turn, send their axons through the anterior olfactory nucleus (AON), and then towards the piriform cortex and the olfactory tubercle in the brain. In the olfactory bulb, the axon terminals of the main excitatory glutamatergic olfactory sensory neurons and the dendrites of mitral/tufted neurons are surrounded by various gamma-aminobutyric acid positive (GABAergic) and dopaminergic inhibitory interneurons, such as periglomerular cells and granule cells.

## 3. The Effects of Essential Oils and Terpenes Through the Olfactory System 

Studies using inhalation of essential oils have shown various benefits that essential oils produce through the olfactory system, as well as differences in their impacts, depending on the types of essential oils. For example, when human subjects were exposed to lavender (*Lavandula angustifolia*) aroma through a diffuser, their working memory decreased and reaction time was slower in tasks that required memory and attention, whereas rosemary (*Rosmarinus officinalis*) aroma enhanced their performances [43]. The essential oils also affected the mood of the subjects. The aroma of rosemary (*Rosmarinus officinalis*) made subjects more alert than the control group and the lavender (*Lavandula angustifolia*) aroma group [43], and more active and ‘fresher’ [44]. Similar differences depending on the type of essential oil were found for peppermint (*Mentha piperita*) and ylang-ylang (*Cananga odorata*) [45]. Peppermint (*Mentha piperita*) enhanced immediate recall of words and alertness, whereas ylang-ylang (*Cananga odorata*) enhanced calmness and made the reaction time longer [45]. These differences suggest that the aroma of lavender (*Lavandula angustifolia*) and ylang-ylang (*Cananga odorata*) would be better to use for relaxation, whereas that of peppermint and rosemary would be useful in enhancing alertness and enhancing memory function. Similar enhancement of cognitive function was also found in the aroma of common sage (*Salvia officinalis*) and Spanish sage (*Salvia lavandulifolia*) [46]. In studies using mice, rosemary (*Rosmarinus officinalis*) suppressed serum corticosterone level, suggesting its anti-stress effect [47]. Further, 2-Phenylethanol, which is a main component of rose (*Rosa*) essential oil, decreased immobility time in a tail suspension test, suggesting its anti-depression effect, but it did not affect cognitive function, activity level, muscle strength, or aggression in mice [48].

The major chemical constituents of common sage (*Salvia officinalis*) are thujone, 1,8-cineole, camphor, carnosic acid, oleanoic acid, ursolic acid, and rosmarinic acid [46]. The major constituents of rosemary (*Rosmarinus officinalis*) are *p*-cymene (44.02%), linalool (20.5%), *γ*-terpinene (16.62%), thymol (1.81%), β-pinene (3.61%), *α*-pinene (2,83%), eucalyptol (2.64%), and β-caryophyllene (0.11%) [49], although there are differences in the constituents depending on where they originated [50] as well as season [51]. For example, 1,8-cineole is not reported as one of the major constituents of rosemary (*Rosmarinus officinalis*) in Turkey [49], but it is one of the major constituents of rosemary (*Rosmarinus officinalis*) in Belgrade, Serbia [51] with seasonal changes, i.e., young to old leaves, from 6.4% (old) to 18.0% (young) [51]. Studies using 1,8-cineole have shown that 1,8-cineole produces a similar impact especially on improving cognitive function but not on mood [52], suggesting that the impact of rosemary (*Rosmarinus officinalis*) on cognitive function and on mood could be mediated by different type of chemical compounds and/or routes. Not only the season or location of origin, but also the mode of production (steam distillation, hydrodistillation, and so on), distillation parameters (temperature, flow, pressure, distillation time, fractional distillation), and other parameters, such as storage condition and age of the essential oil, aging by exposure to oxygen or ultraviolet light can result in different effectiveness [6]. This indicates the importance of knowing the chemical compounds and the amounts included in the essential oils used in these studies, as it is possible that these caused differences in the results among the various published studies. It is also important to test chemical compounds with precisely controlled concentrations, and compare those results with those of tests using complete essential oils to determine the roles of the constituents.

## 4. The Effects of Essential Oils and Terpenes Through the Olfactory Receptors Expressed in the Non-Olfactory System

It has been known from early in the 1990s that olfactory receptors are expressed in non-olfactory tissues, such as sperm cells [53,54,55], testes [55,56], heart [57], kidney [58], skin [59], and gut/intestine [60,61,62]. These olfactory receptors are not involved in sensing odors but are involved in chemical reactions, such as chemotaxis [54], adjusting blood pressure [58], and stimulating secretion of hormones [57] and enzymes [58]. This suggests the possibility that olfactory receptors can be involved when essential oils are topically applied and when they are ingested.

### 4.1. The Skin

**Figure 2:**
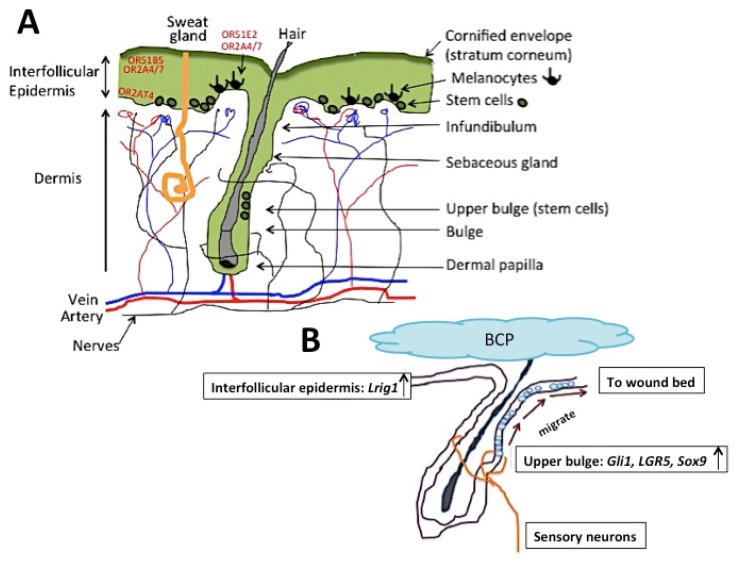
The morphology of skin (**A**) and a hypothetical model on the impact of β-caryophyllene on wound healing in mice (**B**). There are stem cell niches in the basal epidermis layer (shown as “Stem cells” in (**A**)) and in the upper bulge (shown as “Upper bulge (stem cells)” in (**A**)). Locations of human olfactory receptors OR2AT4, OR2AT4/7, OR51B5, and OR51E2 are indicated in red. The stem cells born at the basal epidermis layer differentiate into keratinocytes. The stem cells born at the upper bulge region of hair follicles differentiate into a new hair and do not participate in epidermal homeostasis [64]. When there is a cutaneous wound, the stem cells born at the upper bulge of hair follicles migrate to the basal epidermis stem cell region and convert to epidermal stem cells, and migrate to the wound bed (illustrated in (**B**)) [64]. BCP: β-caryophyllene.

Recent studies have found that human olfactory receptor OR2AT4 is expressed in the epidermis, with the strongest expression in the basal layer, stratum basale (Figure 2A), and that OR2AT4 becomes activated by sandalore, a synthetic sandalwood (*Santalum*) odorant [59]. Activation of OR2AT4 stimulated cell proliferation and migration, activation of cAMP-dependent pathways, and phosphorylation of extracellular signal-regulated kinases (Erk1/2) and p38 mitogen-activated protein kinases (p38 MAPK). This was the first demonstration of an olfactory receptor expressed in human skin that is activated by a well-known aromatic odorant chemical compound, and that has a physiological effect. The authors suggested a possible use of sandalore in improving wound healing. OR2AT4 was later found to be expressed in the outer root sheath of hair follicles. Stimulation of OR2AT4 by sandalore suppressed apoptosis and extended hair growth and longevity [63], which suggests another possible use of OR2AT4, i.e., in avoiding thinning of hair.

Another human olfactory receptor, OR51E2, was found in human epidermal melanocytes [65] (Figure 2A). The ligand of this olfactory receptor was β-ionone, which is an aroma-odorant included in, for example, rose (*Rosa*) essential oils. Activation of OR51E2 by β-ionone stimulated melanin synthesis, suggesting the possibility of utilizing it in the “treatment of pigmentation disorders and proliferative pigment cell disorder such as melanoma” [66].

Human olfactory receptors OR2A4/7 and OR51B5 are also expressed in human skin (Figure 2A) and they are activated by the odorants cyclohexyl salicylate and isononyl alcohol, respectively [67]. Cyclohexyl salicylate has a rather strong flowery fragrance; it is used in, for example, shampoos, shower gels, and soaps. Locations of expression of OR2A4/7 and OR51B5 were slightly different, although both were expressed in the epidermis: OR2A4/7 was expressed in keratinocytes in the supra-basal epidermis (stratum spinosum) and in the melanocytes in the basal layer (stratum basale), whereas OR51B5 was found expressed only in the keratinocytes of the supra-basal layer, but not in the basal layer of the epidermis (Figure 2A). Their impacts were also different. OR2A4/7 stimulated cytokinesis, cell migration, regeneration of keratinocytes, phosphorylation of AKT and Chk-2, and secretion of IL-1, whereas OR51B5 stimulated cell migration, phosphorylation of Hsp27, AMPK1, and p38MAPK, and secretion of IL-6. These studies have shown that, although there are some differences in the locations of expression and functions of these olfactory receptors, all of them similarly stimulate cell proliferation and cell migration when they become activated by ligand aroma-odorants. Figure 2A shows a simplified diagram of the morphology of skin. There are two areas, the basal epidermis and the upper bulge of hair follicles, where stem cells are produced to maintain skin homeostasis and hair regrowth, respectively. The results suggest the possibility that essential oils with chemical compound constituents that are the ligands for these olfactory receptors may become new clinical agents to enhance skin homeostasis and hair regrowth.

Studies using mice have found that the essential oil of oleoresin, *Copaifera paupera*, which contains β-caryophyllene, improves wound healing [68], although it was not clear in that study whether β-caryophyllene produced the impact nor whether an olfactory receptor was involved. Studies using β-caryophyllene (and not essential oils that contain β-caryophyllene) have shown that, topical application of β-caryophyllene on cutaneous wounds can improve re-epithelialization, but the olfactory system was not involved in the impact [2]. As β-caryophyllene activates several different types of receptors other than olfactory receptors, the impact on improving re-epithelialization can be mediated by activating other routes. In these studies [2], although β-caryophyllene is known as a cannabinoid receptor 2 (CB2) ligand [5], the CB2 gene was down-regulated in skin 17 h post-injury+β-caryophyllene-application, whereas transient receptor potential cation channel subfamily melastatin (TRPM1, TRPM6), and transient receptor potential cation channel subfamily vanilloid (TRPV4, TRPV6) were significantly up-regulated [2]. This suggests the possibility of the involvement of these channels in improving wound healing. RNA sequencing and pathway analyses of skin exposed to β-caryophyllene showed that the pathways related to cell proliferation and cell migration were significantly activated (the sonic hedgehog pathway, the planar cell polarity signaling pathway, the fibroblast growth factor signaling pathway, and the Wnt β-catenin signaling pathway) [2]. Studies have found that hedgehog signaling is involved in epidermal homeostasis [69] and that sonic hedgehog secreted from sensory neurons surrounding the hair follicle bulge stimulates Gli1+ (a hair follicle bulge marker) cells in the upper hair follicle bulge to convert to multipotent stem cells, which will migrate to the epidermis and contribute to re-epithelialization [70]. These studies suggest that β-caryophyllene application improves re-epithelialization through enhancing the conversion of hair follicle bulge stem cells into multipotent stem cells, and stimulating their migration towards the basal epidermis, and, from there, towards the wound bed (Figure 2B).

**Figure 3:**
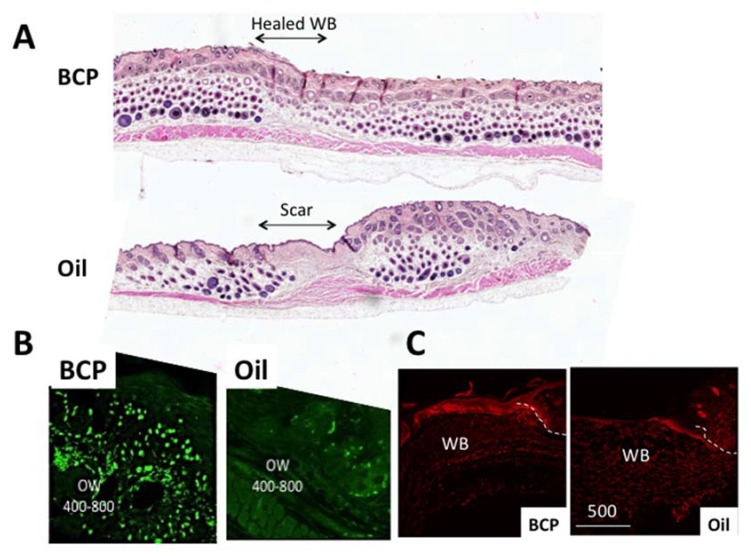
Skin exposed to β-caryophyllene or oil. (**A**) Six weeks after the application of β-caryophyllene for five days. The wound bed (WB) lacks scar formation compared to the clear scar in the oil group control. Hematoxylin and eosin (H&E) staining. (**B**) Cell proliferation (bromodeoxyuridine (BrdU)+ cells) (green) was higher in the dermis and epidermis of skin post-wound day 4 exposed to β-caryophyllene compared to the Oil group control. BrdU was injected twice, every two hours, and the skin was harvested four hours after the first injection. OW 400–800: out of wound bed, 400 to 800 um. (**C**) LGR5 (red) expression was stronger in the skin post-wound day 4 exposed to β-caryophyllene, compared to oil group control (from [2]). The dotted line indicates the wound edge. WB: wound bed.

In the incident that inspired René-Maurice Gattefossé [1] to study the functions of essential oils and the chemical constituents that are involved, the anecdotal story goes that he noticed less scar formation on his burnt hand. In the studies in which β-caryophyllene was topically applied to a cutaneous wound, many genes related to embryonic growth were found up-regulated (cell migration (e.g., Adamts), cell fate determination, and hair follicle formation (*Bambi*, *Msx2*, *Dlx3*, *Padi1*, *Hoxc13*, *S100a*)) [2], suggesting the possibility of more complete skin regeneration. In the field of scar formation and regeneration, embryonic growth is a topic of strong interest because of the more complete regeneration (lack of scars) in injured embryos (personal communication with Dr. Anthony Mescher of Indiana University, School of Medicine who studies regeneration). Figure 3A shows skin, six weeks post-wound, that was exposed to either β-caryophyllene or oil (control) for the first four days (unpublished preliminary data of Koyama, S.). Injured skin exposed to β-caryophyllene shows an extensive number of hair follicles (dark round staining) inside as well as outside of the wound bed (WB) compared to a control group exposed to oil, which clearly shows a scar. Figure 3B shows the proliferating cells, using an injection of bromodeoxyuridine (BrdU), in skin four days post-wound exposed to β-caryophyllene or oil (control), which demonstrates the strong impact of β-caryophyllene on cell proliferation. One of the hair follicle bulge markers, Lgr5 (Leucine-rich repeat-containing G-protein coupled receptor 5), also showed stronger expression in the epidermal cells and dermal cells migrating toward the center of the WB in skin exposed to β-caryophyllene compared to oil controls (Figure 3C).

### 4.2. The Gut and Intestine

**Figure 4:**
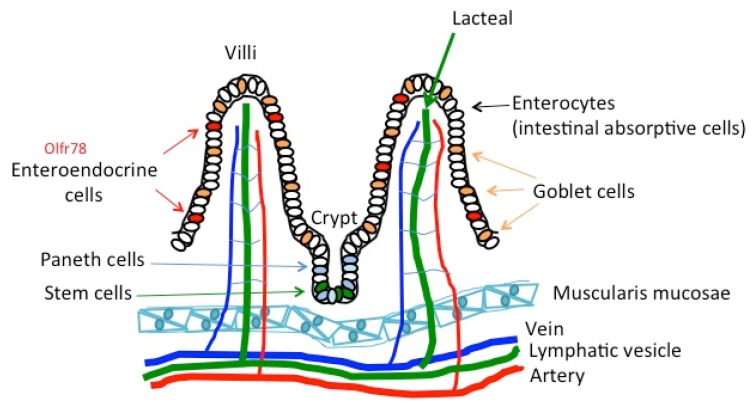
The morphology of villi in the intestine. Olfactory receptor Olfr78 is shown in red. Other olfactory receptors that have been found are not indicated, as the specific cell types are not determined yet.

Similar to the skin, the epithelium of the intestine serves as a barrier, or provides host-immunity, between the internal and external environment, in addition to its function to absorb nutrients. Figure 4 shows the morphology of the villi of the intestine. A region called the crypt at the bottom of the villi possesses stem cells that function to maintain the villi. Different from the skin, the epithelium of the intestine has a monolayer with different types of cells on the same surface (Figure 4).

Recent studies have found that the chemical compounds in orange essential oils, i.e., limonene, linalool, and citral, enhance the microbiota, especially *Lactobacillus*, in the gut of mice, and limonene had the strongest impact [71]. The olfactory receptor for limonene, Olfr56 (human homolog, OR2V1) [72,73,74,75], is expressed in both the large intestine as well as in the small intestine in mice. Olfactory receptor Olfr78 (human homolog, OR51E2) was also found expressed in some, but not all, of the enteroendocrine cells in the colon of mice, and propionate was found as their ligand [61]. In a separate study, Olfr78 responded to acetate and propionate [58]. Propionate is an important nutrient generated by bacteria, which stimulates secretion of satiety-inducing hormones that can adjust energy homeostasis [76]. These studies suggest that essential oils may be involved in regulating energy homeostasis by enhancing the microbiota, which will stimulate the secretion of propionate, which is the ligand of olfactory receptors. In the studies on Olfr78 in the small intestine, activation of Olfr78 was involved in regulating blood pressure as well, which also suggests the role of olfactory receptors in adjusting homeostasis. Since propionate is a ligand of Olfr78, there is a possible role of essential oils in regulating blood pressure [58]. Studies have also indicated that geraniol, a monoterpene found in various flowers (rose (*Rosa*), lemon (*Citrus limon*), lavender (*Lavandula angustifolia*)) and fruits, and citronellal, which is a monoterpenoid with a strong lemon-like smell, stimulate glucagon-like peptide 1 secretion in mouse intestinal tissues and in cultured enteroendocrine cells [62].

In a study using mice, in which geraniol was intraperitoneally injected (thus, not through the digestive system) and responses to acetic acid were measured, geraniol was found to have as strong an analgesic impact as morphine, measured by the latency, to show writhing after intraperitoneal injection of acetic acid [77]. Oral treatment of geraniol in the same study did not produce as strong an impact as intraperitoneal injection of geraniol in the latency to show writhing, but the number of writhing events was less than the control group, which did not receive geraniol nor morphine treatment before injection of acetic acid [77]. In a separate study, orally administered geraniol was found to have an anti-depressant impact, measured by the immobility time in forced swimming tests and tail suspension tests [78]. Corticosterone level was also significantly lower in the mice that received geraniol [78]. These studies suggested that geraniol may be used in analgesic treatments, instead of using opioids or allowing decreased use of opioids. Chirumbolo and Bjorklund [79] suggested a role of glutamatergic neurotransmission and transient receptor potential cation channels (TRP channels) in these actions. In addition, monotremes with chemical kinships, e.g., geraniol, limonene, *α*-phellandrene, and the carvones may similarly have anti-nociceptive action. It is possible that these compounds are ligands of the same receptors and have similar effects.

Studies using human subjects (9 males and 15 females, ages 21 to 35, mean age 25.2) utilized a method in which subjects consumed one of two types of capsules, which contained either 500 uL of peppermint (*Mentha piperita*) essential oil, or vegetable oil. All subjects drank 200 mL of milk one hour before the tests started [80]. The peppermint (*Mentha piperita*) contained, among others, 1,8-ceneole (5.34%), limonene (2.06%), linalool (0.47%), *α*-pinene (0.42%), β-pinene (0.71%), and *α*-thujene (0.02%). This method of taking a capsule is beneficial in isolating the impact of the digestive system and reducing the influences of other routes to a minimum. A small amount of the aroma-odorants may still come up from the stomach when the odorants are released from the capsule. In these studies, the subjects who took essential oils showed less fatigue to cognitively demanding tasks and showed higher cognitive function [80].

## 5. The Effects of Essential Oils and Terpenes through Non-Olfactory Receptors

Odors activate not only olfactory receptors but also non-olfactory receptors. As mentioned above, studies have shown that a gavage of β-caryophyllene activated CB2 [5] and topical application of it up-regulated TRPM1, TRPM6, TRPV4, and TRPV6 of the TRP channels [2]. These studies indicate that β-caryophyllene activates at least three types of receptors: olfactory receptors, TRPs, and CB2. Recent studies have also shown that TRP channels become activated by some phytochemicals [81], indicating that β-caryophyllene is not the only ligand that activates TRP channels. A growing number of studies show roles of TRP channels in the initiation of pain and itch perception as well as epidermal homeostasis and hair follicle regulation in skin, which makes TRP channels the ‘ionotropic cannabinoid receptors’. These studies suggest the possibility that multiple channels could be involved in mediating the effects of essential oils.

The skin is comprised of multiple cell layers, which is different from the olfactory epithelium (pseudostratified ciliated columnar epithelium) and gastro-intestinal system (mostly simple columnar epithelium). As written above, the olfactory receptors expressed in skin are expressed in multiple types of cells in the epidermis. The CB2 receptor is expressed in nerve cells, immune tissue, hair follicles, sebaceous glands, the dermo-muscular layer in the dermis, and vascular smooth muscle in intact skin [82,83]. TRP channels are expressed in sebaceous glands, keratinocytes, and melanocytes [84]. The locations where the receptors and channels are expressed in the skin are not necessarily exposed to the outer environment. In addition, one of the major functions of skin is to protect the organism by functioning as a barrier. The stratum corneum is the outermost layer of the epidermis with insoluble transglutaminase-cross-linked proteins and lipids, and is considered to function as a barrier [85,86]. It is highly selective and allows only small lipophilic molecules to pass through [87]. This brings up a new hypothesis: differences may exist in the accessibility to the receptors, depending on the permeability of the chemicals. Studies have shown that several methods enhance permeability (using external energy sources, such as ionophoresis, or using chemical permeation enhancers). Chemicals that enhance permeation are fatty acids, fatty alcohols, alcohols and glycols, and terpenes, i.e., terpenes possess the ability to permeate the stratum corneum of skin. Smaller terpenes are better at permeating the skin [88,89]. Other studies indicate that eucalypts (*Euclayptus*), goosefoots (*Chenopodium*), and ylang-ylang (*Cananga odorata*) have been used as chemical permeability enhancers for 5-flouorouracil, a medicine to treat cancer. The principal element in eucalyptus is 1,8-Cineole, and it has a high efficacy in enhancing permeability (pretreatment of human epidermis with 1,8-cineole enhanced the permeability of the drug 100 times) [90]. This indicates not only that essential oils can permeate skin but they can also be used to enhance drug delivery.

## 6. Psychological and Physiological Impact of Essential Oils

The impacts of exposure to essential oils can be classified into psychological impacts and physiological impacts. The psychological impacts found in non-human animals, mostly using mice or rats, are anxiolytic effects, suppression of depression, anti-agitation, as a relaxant, and anti-stress effects [91], which may all be related to a relaxing effect through suppression of anxiety, stress, agitation, and depression. Some studies suggest that English lavender (*Lavendula angustifolia*) has the strongest anxiolytic effect [92]. The physiological impacts of essential oils are broader, i.e., improving wound healing, as an analgesic, an anti-nociceptive, an anti-fungal, an anti-inflammatory, an anti-oxidant, a local anesthesia, a sedative, an anesthetic systemic, having anti-convulsant effects, and others [91,92] (Table 2). Studies using human subjects have found differences in effectiveness, depending on the essential oils as shown above. Exposure to aroma odorants is done mostly through inhalation methods. Some studies used drinking or swallowing of capsules to confine the exposure mostly to the gastro-intestinal system (Table 2).

These differences in the application methods can produce differences in the effects because, as written in Section 2, environmental conditions largely affect sensitivity to odors. Evaluations of the response are conducted mostly by assigning tasks or surveys to subjects, while some studies use electroencephalography (EEG) to record the responses to the odors [93]. In the studies using EEGs, it was found that lavender (*Lavandula angustifolia*), eugenol, or camomile (*Asteraceae*) aroma inhalation significantly reduced α1 waves (8–10 Hz) at the parietal and posterior temporal regions, suggesting a decrease in arousal level [93]. Another study found that rosemary (*Rosmarinus officinalis*) decreases frontal α and β activity, suggesting increased alertness [93].

Although there are some essential oils with rather small numbers of chemical compound constituents (for example, about 25 in the case of guaicawood (*Bulnesia sarmienti*) oil), most essential oils have 100 to 250 different chemical compound constituents, and some contain 300 to 400 [6]. In the very popular lavender (*Lavandula angustifolia*) and rosemary (*Rosmarinus officinalis*) oils, studies have identified 505 and 450 chemical compounds, respectively [6]. Chemical compounds that constitute more than 50% of the oil are called major constituents [6]. It would be reasonable to test these major chemical compounds, compare the effect of these essential oils, and then test minor chemical compounds to determine their effects as well. Some chemical compounds are common in many essential oils. These are limonene, linalool, *α*-pinene, β-pinene, β-caryophyllene, myrcene, 1,8-cineol, sabinene, geraniol, *α*-terpineol, *p*-cymene, linalyl acetate, and *γ*-terpinene [6]. Table 3 summarizes the major constituents of some of the essential oils from Table 2 and some additional types of oils. Comparison of the major constituents of the same essential oil in several studies shows that the chemical compounds listed as major constituents vary. The amount of these major constituents varies depending on the part of the plant used, e.g., old leaves versus young leaves, different seasons, geographical locations, and even the weather depending on the year. This makes it critical to measure the constituents of the essential oils used in experiments, to control the exposure methods (inhalation vs. capsule digestion vs. topical application) as well as to state the environmental conditions, which can affect the exposure (temperature, humidity, air flow). The data also indicate the importance of testing the effects of pure single chemical compounds contained in essential oils. This will make experiments comparable and help to determine the influences of each chemical compound and the concentration dependency of its effect.

An increasing number of studies have used pure chemical constituents of essential oils to test their effects. Table 4 summarizes some of the results of these studies, which tested the impact at the chemical compound level (Table 4).

As we can see in the table, many chemical compounds produce similar effects and some produce opposite effects. The similar effects may be due to activation of the same receptors by these different chemical compounds. There are anti-inflammatory impacts, cell viability, and proliferation stimulation or suppression, and many chemical compounds have anxiolytic or anesthetic effects. These impacts are anti-cancer, anti-parasitic, and antiseptic in nature. Many chemical compounds enhance or suppress cognitive function and affect hormone secretion. The effects on brain activity and hormone secretion could be mediated by the olfactory system because activation of olfactory bulb neurons is transmitted not only to the olfactory cortex but also to the hypothalamus and amygdala areas that are involved in the control of hormone secretion [186]. Possibly, effects on brain and hormone secretion are mediated by the olfactory system, whereas other effects, such as changes in cell viability, proliferation, and pain are mediated by other receptors.

## 7. The Receptors and Neuromodulators 

As summarized in Table 4, many aromatic chemical compounds have anti-inflammatory and analgesic influences. This suggests that these chemical compounds activate the same signaling pathway. In our previous study [91], we summarized the receptors and channels that some of the aromatic chemical compounds activate and showed the chemical compounds with similar impacts. For analgesic properties, 1,8-cineole, menthol (both (-)- and (+)-), carvone (both (-)- and (+)-), pulegone, linalyl acetate, linalool, carvacrol, estragole, besabololl, carvone (both (-)- and (+)-), terpinen-4-ol, which are all monoterpenes, are known to have analgesic properties targeting Na^+^ and TRP channels [91]. TRPV1 to 4 are temperature-sensitive channels activated by heat stimuli, whereas TRPM8 and TRPA1 are temperature-sensitive channels that are activated by cold stimuli [187]. Recent studies on aroma chemical compounds showed that some of them are agonists of these channels while some are antagonists. 1,8-Cineole affects Na^+^ channels [188] and functions as a TRPM8 agonist and a TRPA1 antagonist [189]. Menthol (chemical compound made synthetically from various mints (*Mentha*)), which is well-known in producing a cooling effect, is a TRPM8 agonist [190]. The cooling effect can be explained as a cold hyperalgesia caused by activating TRPM8 channels and suppressing compound action potentials (CAP), raising the temperature threshold to feel coldness, i.e., making the sensation of coldness occur at a higher temperature. TRPV1 is one of the temperature-sensitive channels specific for heat stimuli (>43 °C) and extracellular pH, and it also has a crucial role in pathological forms of pain [191,192]. Activation of TRPV1 induces pain hypersensitivity (thermal hyperalgesia).

There are two types of GABA receptors, GABA_A_ are GABA-gated chloride channels located in post-synaptic membranes, whereas GABA_B_ are G-protein coupled receptors located both in pre- and post-synaptic membranes [193]. There are also two sub-types of GABA_B_, which are called GABA_B1_ and GABA_B2_, and there are GABAa-rho receptors, which form distinct ligand-gated Cl^−−^ channels (previously called GABA_C_) [194]. Activation of the GABA_B_ receptor was found to inhibit TRPV1 sensitization, and TRPV1 activation triggers GABA release [191]. Importantly, this inhibition of TRPV1 through activation of GABA_B_ receptors selectively suppresses the sensitized status of TRPV1 and does not affect acute TRPV1 pain signaling [191,192]. There are many plants (for example green tea (*Camellia sineusis*) and soybean (*Glycine max*)), which include GABA [194,195,196] and there are many chemical compounds in plants, which modulate the action of GABA on GABA receptors. Modulation of action means enhancing or suppressing the action of GABA on GABA receptors. These compounds are called positive modulators and negative modulators, respectively. For example, some monoterpenes such as thymol, thymoquinone, and borneol, are known as positive modulators for GABA_A_ receptors, whereas alpha-thujone acts as a negative modulator of GABA_A_ receptors and as an antagonist of 5-hydroxytryptamine (5HT_3_) receptors. Sesquiterpenes bilobalide and picrotoxinin are also negative modulators, whereas valerenic acid and isocurcumenol are positive modulators of GABA_A_ receptors [196]. Often multiple modulators are included in essential oils, and some modulators serve as secondary modulators, which function in conjunction with other specific modulators and enhance the action of these other modulators [196]. While it is important to test single compounds to determine the effects of essential oils on the one hand, on the other hand, the effects of essential oils need to be tested with multiple chemical compounds in the assay to determine the possibility of synergistic effects.

GABA is known as an inhibitory neurotransmitter [193]. Studies have shown that GABA has a neuroprotective effect against degeneration, preventing neurotoxic-induced cell death, decreasing apoptosis, and suppressing cytodestructive autophagy [193]. Some studies have also reported that GABA can improve memory and cognitive function, shorten sleep latency, and increase non-rapid eye movement sleep time, prevent depression, improve long-term memory, and increase neuronal cell proliferation, down-regulate hypertension and decrease blood pressure [91,193]. GABA is also known to have an anti-diabetic effect, anti-cancer effect, anti-oxidant effect, anti-inflammatory effect, anti-nociceptive effect, anti-microbial effect, and anti-allergic effect [91,193]. These studies suggest that it is highly likely that the GABA system and TRP channels have crucial roles in the mechanisms that mediate the effects of chemical compounds in essential oils.

Recent studies have shown that CB2 receptors are also activated by one of the aroma chemical compounds, β-caryophyllene [5], even though there are some negative reports [197]. These studies have provoked a strong interest toward β-caryophyllene among neuroscientists who work on the endocannabinoid system, and produced a big trend to study the impact of β-caryophyllene. Such strong interest generated both positive and negative impacts on scientists who are not familiar with essential oils. As a positive impact, for example, scientists started to show more interests in phytochemicals and essential oils, which have often received little interest and many scientists had even skeptical images of their effects, especially in western culture. As a negative impact, although essential oils contain many aromatic chemical compounds that have been known to have analgesic and anti-inflammatory impacts, many scientists, especially, most likely those who are not familiar with essential oils, tended to simply think that β-caryophyllene was the chemical compound that produced the effect, when an essential oil containing β-caryophyllene showed an impact, expressing it in a way like “the essential oil worked *because it contains* β*-caryophyllene*”. It is necessary to consider that some essential oils contain 400 different constituents and many terpenes have analgesic impacts and anti-inflammatory impacts.

β-caryophyllene has anti-inflammatory and analgesic effects [5,198], again contradicted by some negative reports [199]. These discrepancies may be due to several factors. For example, the effects of α-pinenes vary depending on the monoterpenes and sesquiterpenes in the environment. Synergetic effects and oxidation states of these compounds have been established [200]. Although many terpenes are anti-oxidants, it has been suggested that this anti-oxidant property functions only in a lipophilic environment [200,201]. Another possibility is that many of the ligands of TRP channels activate cannabinoid receptors as well [202]. These results show again the complexity of the mechanisms of action and that multiple routes are involved regarding the effects of terpenes.

In the case of the olfactory system, the signaling pathway starts with activation of olfactory receptor proteins in olfactory sensory neurons, which send the signal to the olfactory bulb and then to various locations in the brain [203,204]. Signaling from the accessory olfactory system and main olfactory system reaches separate locations in the brain. Signals processed in the main olfactory system reach the hypothalamus from the piriform cortex, whereas signals processed in the accessory olfactory system reach the hypothalamus from the medial amygdala. Generally speaking, the accessory olfactory pathway used to be known as the pheromone signaling pathway, but it is now known that the receptors for pheromones are expressed in the main olfactory system and in some of the olfactory subsystems. The accessory olfactory pathway was considered the pathway that affects hormone secretion because the signaling reaches the amygdala and hypothalamus, but it is also now known that some portion of the signaling in the main olfactory pathway reaches the areas in the brain that regulate hormone secretion and there are feedback loops as well [186]. This suggests that responses in the brain will depend on the location of olfactory receptors and, subsequently, where the signals are being sent in the brain. Lavender (*Lavandula angustifolia*) is known to suppress cognitive function, whereas rosemary (*Rosmarinus officinalis*) enhances it [43]. This suggests that some constituents of these essential oils activate olfactory signaling with a different contribution of neuromodulators. Testing each constituent will determine the role of each constituent, and how it generates the opposite physiological response.

## 8. Conclusions

As we summarized in this review, studying the effects of essential oils and terpenes requires interdisciplinary approaches because of the multiple methods of their use: diffusers, which will use the olfactory system primarily; drinking, which will use primarily the digestive system; and topical application, which will enter primarily through the skin. Experimental techniques in olfactory neuroscience, skin biology, and small intestine physiology will allow testing the effects of the essential oil constituents, which are likely to be different depending on the application routes. The vast number and types of herbal plants necessitates interaction with plant biologists. Handling the constituents of essential oils requires knowledge and techniques in analytical chemistry.

The long history of using essential oils and the fact that they were often used in religious ceremonies, and for relaxation, has contributed to our images of mysterious or “non-scientific” substances. We now have more scientific evidence on, e.g., the anti-inflammatory, analgesic, and anti-microbial effects at the chemical compound level compared to the effects of complete essential oils. Understanding the effects of each constituent and combinations of constituents in in vivo and in vitro studies may lead to the development of new medical agents for wound healing, bacterial infection, and healing of other diseases, including cancer. It is time to thoroughly test and determine the large potential of terpenes in essential oils in order to improve health conditions because of their impacts on the olfactory system, the skin, and the gastro-intestinal system.

## Figures and Tables

**Table 1:** Clusters and odors related to each cluster (Domain classification is based on Mori and Sakano [31]; summarized using Mori et al. [30] and Mori and Sakano [31]).

Domain	Cluster	Examples of the Odorous Chemical Compounds, which Activated the Cluster	Odor Quality
Dorsal (DI)	A	Fatty acids and alkylamines [31]. Aliphatic acids, aliphatic aldehydes as well as “the subsets of these odorants with a similar carbon chain length” [30]. Diketones, benzaldehyde, and benzoic acid; common features are “a carboxyl group (-COOH), a diketone group (-(CO)(CO)-), or an ester group (-COO-)”.	Fatty Rancid sour pungent
Dorsal (DII)	B	Aliphatic alcohols, aliphatic ketones, and phenyl ethers [31]. “Aliphatic alcohols with relatively long carbon chain and to a wide range of aliphatic ketones”. Subsets of esters. Anisole and its derivatives with a methoxy group (-O-CH_3_). Combination “of elongated carbon chain structures with a hydroxyl group (-OH), an alkoxyl group (-O-R), or a carbonyl group (>C=O)”.	Floral Fruity Green and grassy Anisic
Dorsal DII	C	Phenols and phenyl ethers [31]. “Phenol family odorants, molecules having a hydroxyl group attached to the benzene ring”. Phenyl ethers; “molecular features of a benzene ring with hydroxyl group, a methoxy group, or an ethoxy group”.	Phenolic Medicinal Spicy Ethereal
	D	Aliphatic and aromatic ketones [31]; “responses to a variety of ketones: aliphatic ketones, aliphatic-aromatic ketones, diketones, and cyclic ketones”.	Spicy Minty
Lateral/ventral	E	Not specified in Mori et al. [30] nor in Mori and Sakano [31]; weak responses to ethers [30].	
	F	Aliphatic “ketones, phenyl ethers, diketones, aliphatic-aromatic ketones, and cyclic ketones”.	
	G	Phenyl “ethers, diketones, aliphatic ketones with relatively short side chains, aliphatic-aromatic ketones, cyclic ketones and ethers”.	
	H	“Benzene-family odorants”. No response “or only a weak response to cyclic terpene hydrocarbons”. Some “respond to open-chain hydrocarbons”. “benzene derivatives with a polar functional group, such as anisole”, phenol, benzaldehyde, or acetophenone.	Gassy and kerosene-like odor
	I	“respond to at least one of the cyclic terpene hydrocarbons”. Subset of benzene-family odorants; cyclic terpene ketones, cyclic terpene alcohols	Citrusy Woody
Dorsal DII	J	Thiazoles and thiazolines [31].	

*: Cited parts (““) are from Mori et al. [30].

**Table 2:** Impact of essential oils and the methods of application.

Latin Name	Plants Common Name	Species	Routes Used in Experiments	Results	References
*Achillea wilhelmsii*	Achillea	Rat	IP; gavage	Anxiolytic effect; improved healing of gastric ulcer; anti-hypercholesterolemic	[94,95,96]
*Acorus gramineus*	Grass-leaf sweet flag	Mice; rats; bacteria	Inhalation; gavage; in vitro	Anti-convulsive; sedative effect; act on central nervous system via GABA system; anti-depression; anti-epilepsy through GABA system; anti-fungal	[97,98,99,100]
*Acorus tatarinowii*	Acorus	Mice	IP; gavage	Anti-depressant; enhanced serotonin transporter	[101,102]
*Anethum graveolens*	Dill	Mice	Topical	Improved infected wound healing; anti-oxidant	[103,104]
*Annona vepretorum*	Anaticum	Mice	Gavage	Anti-oxidant; Anti-convulsant; sedative; anxiolytic; anti-depressant	[105,106]
*Artemisia herba-alba*	White wormwood	Bacteria	In vitro	Anti-bacterial activity; anti-fungal; anti-oxidant, anti-cancer, anti-bacterial; anti-inflammatory	[107,108,109]
*Artemisia ludoviciana*	Silver wormwood	Mice	IP	Anti-nociceptive	[110]
*Artemisia judaica*	Judean wormwood	Yeasts	In vitro	Anti-oxidant; Anti-fungal; anti-biofilm; anti-inflammatory	[111,112]
*Artemisia dracunculus*	Tarragon	Rats; Mice	IP	Anti-convulsant; Anti-nociceptive effect; anti-inflammatory effect	[113,114,115]
*Asarum heterotropoides*	Asarum	Mice	Inhalation	Anti-depressant; anti-bacterial	[116,117]
*Camellia sinensis*	Tea tree; tea shrub	Mice	Inhalation	GABA-A enhanced using oolong tea; anti-oxidant using green tea	[118,119]
*Cananga odorata*	Ylang-ylang	Human	Inhalation	Decreased arousal level; effective in treating depression, high blood pressure, and anxiety. Anti-microbial, anti-biofilm, anti-inflammatory, anti-vector, insect-repellent, anti-diabetic, anti-fertility, anti-melanogenesis	[45,120]
*Cinnamon verum*	Cinnamon	Mice	Topical	Improved infected wound healing	[121]
*Citrus aurantium, L.*	Bitter orange	Mice	Gavage	Anxiolytic effect mediated by 5-HT (1A) receptors; reduce cholesterol; larvicidal effect	[122,123,124]
*Citrus bergamia*	Bergamot orange	Mice; rat		Decreased stress-induced anxiety; anxiolytic, sedative; suppress pain	[125,126,127,128]
*Citrus sinensis*	Sweet orange	Mice; rat; human		Anxiolytic effect; nitrogenic neurotransmission plays a role	[129,130,131]
*Copaifera paupera*	Oleoresin	Mice	Topical application	Improved wound healing; anti-leishmanial	[68,132]
*Coriandrum sativum, L.*	Coriander	Rat	Inhalation	Anxiolytic effect; cognitive enhancement; anti-bacterial, anti-fungal, anti-oxidative effect	[133,134,135]
*Cymbopogon citratus*	Lemon grass	Mice	Gavage; IP	Anxiolytic effect through GABA system; increased sleeping time; anti-convulsive effect at least partially mediated by GABA system; cholesterol reduction; anti-amoebic, anti-bacterial, anti-diarrheal, anti-filarial, anti-fungal, anti-inflammatory, anti-malarial, anti-mutagenicity, anti-mycobacterial, anti-oxidants, and hypoglycemic and neurobehavioral effects	[136,137,138,139,140]
*Cymbopogon winterianus*	Citronella grass	Mice	IP	Anti-convulsant; Molluschicidal and larvicidal activity	[137,141,142]
*Dysphania graveolens*	Fetid goosefoot	Mice	Gavage	Anti-nociceptive, analgesic effect not through 5-HT2A/2C	[143]
*Hyptis* *Mutabilis*	Tropical bushmint	Silver catfish	In water	Sedative effect not through GABA system	[144]
*Lavandula angustifolia*	English lavender	Human; Mice; in vitro study with rat cells	Inhalation	Decreased cognitive function, decreased arousal level; inhibited TBPS binding to rat GABA-A receptor; anxiolytic-like effect not through GABA system; anosmia does not impair anxiolytic effect; pain suppression; reduced agitation and physical non-aggressive behavior in older people with dementia	[43,144,145,146,147,148,149]
*Lippia alba*	Bushy lippia	Silver catfish	In water	Anesthetic effect through GABA system; S-(+)-linalool in lippia has sedative and anesthetic effect; citral and linalool in it have anesthetic effect	[150,151,152,153]
*Matricaria chamomilla*	Chamomile	Human; review of animal studies	Inhalation; capsule consumption	Anxiety suppression; anti-inflammatory effect, anti-mutagenic effect, cholesterol-reduction effect, anti-spasmotic effect, anxiolytic effect	[154,155,156]
*Melissa officinalis, L.*	Common balm; lemon balm	Rat; human	In vitro; inhalation	Anti-agitation; anti-microbial	[144,149,157,158]
*Mentha x piperita*	Peppermint	Human	Inhalation; digested capsules	Increased arousal level Showed less fatigue to cognitively demanding tasks and showed higher cognitive function; anti-oxidant	[45,80,159]
*Nigella sativa, L.*	Black cumin	Mice	Inhalation	Anti-convulsant effect	[160]
*Piper guineense*	Pepper	Mice	Inhalation; IP	Sedative effect; anxiolytic effect; central nervous system depressant; rectal temperature suppressed, muscle relaxant, induced catalepsy; anti-psychotic effect, anti-convulsant effect	[161,162]
*Rosmarinus officinalis*	Rosemary	Mice; human	Inhalation; topical application	Suppressed serum corticosterone, brain dopamine increased; anxiolytic effect, Increased arousal level More active and fresher feeling; blood pressure enhanced, heart rate enhanced, improved infected wound healing; enhanced cognitive function in humans	[43,44,47,163]
*Salvia lavandulifolia*	Spanish sage	Human	Drink	Enhanced cognitive function	[46]
*Salvia officinalis*	Common sage	Human	Drink	Enhanced cognitive function	[46]
*Salvia sclarea*	Clary sage	Rat	IP	Anti-depressant; anti-stressor; modulation of dopamine	[164]
*Syzygium aromaticum*	Clove		In vitro	Anti-oxidant; anti-bacterial effect; anti-cancer effect	[165,166]
*Tagetes minuta, L.*	Black mint	Chicks	SC	Anxiolytic	[167]
*Thymus capitatus*	Spanish oregano	Mice; rats	Gavage	Anti-nociception; anti-microbial	[168,169]
*Valeriana officinalis, L.*	Valerian	Review		Sedative effect; suppressed GABA breakdown	[170]

SC: subcutaneous, IP: intraperitoneal injection, TBPS: *tert*-butylbicyclophosphorothionate, 5HT: 5-hydroxytryptamine serotonin receptor, 2A, 2B, 2C are subtypes, GABA: gamma-aminobutyric acid

**Table 3:** Essential oils and their major constituents.

Latin Name	Plants Common Name	Major constituents	References
*Achillea wilhelmsii*	Achillea	Luteolin, apigenin, caffeic acid, ferulic acid, leucodin, 1β-epoxydesacetoxymatricarin, 10β-epoxydesacetoxymatricarin, 2-(3,4-dimethoxyphenyl)-5-hydroxy-6,7-dimethoxychromen-4-one, 2-(3,4-dimethoxyphenyl)-5,6,7-dimethoxychromen-4-one, salvigenin	[95]
*Acorus gramineus*	Grass-leaf sweet flag	From rhizomes: *α*-asarone (17.7%), *cis*-asarone (7.29%), asaronaldehyde (5.35%), borneol (2.18%), *γ*-cadinene (2.56%), calarene (1.64%), camphene (0.73%), camphor (3.63%), elemicin (1.98%), euasarone (12.7%), *α*-gurjunene (1.21%), 1,2,4-methenoazulene (0.82%), methyleugenol (34.18%), methyl isoeugenol (4.90%), and *α*-muurolene (0.76%).	[98]
*Acorus tatarinowii*	Acorus	β-asarone, *α*-asarone, veratric acid, anisic acid, 3,4,5-trimethoxybenzoic acid, *trans*-isoferulic acid, 2,4,5-trimethoxybenzoic acid, 4-hydroxybenzoic acid, syringic acid	[102]
*Anethum graveolens*	Dill	Kazemi: *α*-phellandrene (19.12%), limonene (26.34%), dill ether (15.23%), sabinene (11.34%), n-nonadecane (1%), n-eicosane (0.78%), n-heneicosane (0.67%), β-myrcene (0.23%), *α*-thujene (0.21%); Kaur et al.: Carvone, limonene, camphor, carveol, perillyl alcohol	[104,171]
*Annona vepretorum*	Anaticum	Spathulenol (43.7%), limonene (20.5%), caryophyllene oxide (8.1%), *α*-pinene (5.5%)	[105]
*Artemisia herba-alba*	White wormwood	Bourgou et al.: *c*-glycosylated and methylated flavones, quinic acid derivatives, coumarins, sesquiterpenes lactones, terpenoids, fatty acids, carbohydrates, organic acids and alkaloids, di-O-caffeoylquinic acids, artemisinic acid, menthol, *α*-ketoglutaric acid, scopolin, isoschaftoside and sucrose; Mohamed et al.: 1,3,8-trihydroxyeudesm-4-en-7*α*,11βH-12,6*α*-olide, 5-β-_D_-glucopyranosyloxy-7-methoxy-6H-benzopyran-2-one, 3*α*,8β-dihydroxygermacr-4,9(10)-dien-7β,11*α*H,12,6*α*-olide, 1β,8*α*-dihydroxy-11*α*,13-dihydrobalchanin, 11-epiartapshin, tomenin benzoic acid, *p*-(β-_D_-glucopyranosyloxy)-methyl ester	[108,109]
*Artemisia ludoviciana*	Silver wormwood	(±)-camphor, *γ*-terpineol, 1,8-cineole, and borneol	[110]
*Artemisia judaica*		Piperitone (45.0%), *trans*-ethyl cinnamate (20.8%), ethyl-3-phenyl propionate (11.0%), spathulenol (6.27%), *cis*-ethyl cinnamate (5.64%), 2,6-dimethyl phenole (1.39%), methyl cinnamate (1.06%), 2,6-dimethyl phenol (1.39%), camphor (0.38%)	[111]
*Artemisia dracunculus*	Tarragon	*trans*-anethole (21.1%), *α*-*trans-*ocimene (20.6%), limonene (12.4%), *α*-pinene (5.1%), allo ocimene (4.8%), methyl eugenol (2.2%), β-pinene (0.8%), *α*-terpinolene (0.5%), bornyl acetate (0.5%), and bicyclogermacrene (0.5%)	[113]
*Asarum heterotropoids*	Asarum	Methyl eugenol (37.6%), sesamin (22.1%), safrole (14.7%), N-isobutyl-(2E,4Z,8Z,10E)-dodecatetraenamide (8.6%), linoleic acid (3.2%), trans-isocroweacin (5.5%), pentadecane (4.2%), 3,4-benzocyclodec-3-ene-1,5-diyn-7-one (3%), myristicin (1%)	[117]
*Camellia sineusis*	Green tea	Theanine, theobromine, caffeine, gallic acid, (+)-gallocatechin, caffeine, (-)-epigallocatechin, (+)-catechin, (-)-epicatechin, (-)-epigallocatechin gallate, (+)-gallocatechin gallate, (-)-epicatechin gallate, (+)-catechin gallate	[119]
*Cananga odorata*	Ylang-ylang	*p*-methylanisole, methyl benzoate, benzyl benzoate, benzyl acetate, geranyl acetate, cinnamyl acetate, (E,E)-farnesyl acetate, linalool, geraniol, benzyl salicylate, germacrene D, β-caryophyllene, gamma-muurolene, (E,E)-farnesyl acetate	[120]
		Linalool, germacrene D, benzyl acetate, *p*-cresyl methyl ether, (E,E)-*α*-farnesene, geranyl acetate, methyl benzoate, β-caryophyllene	[6]
*Cinnamomum verum*	Cinnamon	Eugenol (17.32%), Benzyl benzoate (0.22%), (E)-Cinnamyl alcohol (0.09%), eugenyl acetate (1.29%), (E)-cinnamyl acetate (11.78%), (Z)-cinnamyl acetate (0.01%), (E)-cinnamaldehyde (33.04%), safrole (0.01%), geraniol (0.05%), piperitone (0.16%), α-terpineol (0.61%), terpinen-4-ol (0.13%), linalyl acetate (2.25%), linalool (16.85%), *p*-cymene (0.92%), *trans*- β-ocimene (0.05%), cis-β-ocimene (0.03%), 1,8-coneole (0.46%), limonene (0.41%), *α*-terminene (0.09%), myrcene (1.17%), sabinene (0.06%), β-pinene (0.19%), camphene (0.09%)	[172]
*Citrus aurantium, L.*	Bitter orange	DI-limonene (94.81%), *α*-pinene (0.30%), β-pinene (0.65%), β-myrcene (1.00%), *trans*-ocimene (0.19%), *γ*-terpinene (0.01%), linalool oxide (0.04%), 1-octanol (0.13%), *trans*-linalool oxie (0.02%), isoterpinolene (0.01%), nonanal (0.02%), *α*-terminolene (0.44%), linalyl acetate (0.32%), sabinene hydrate acetate (0.93%),	[124]
*Citrus bergamia*	Bergamot orange	Limonene (25.62 – 52.19%), linalool (1.75–20.26%), linalyl acetate (15.61–40.37%),	[127]
*Citrus sinensis*	Sweet orange	Limonene (93.5%), β-pinene (2.979%), *α*-pinene (0.792%), *cis*-ocimene (0.235%), linalook (0.206%), *α*-terpineol (0.142%), (E)-citral (geranial) (0.150%), *α*-bergamotene (0.448%), β-bisabolene (0.365%)	[173]
*Copaifera paupera*	Oleoresin	*α*-copaene (A 21.8%, B 38.8%), *trans*-caryophyllene (A 4.1%, B 21.4%), *γ*-cadinene (B 7.7%), caryophyllene oxide (A 12.5%), diterpene kaurene (A 33.2%, B 2.4%)	[132]
*Coriandrum sativum, L.*	Coriander	Linalool (60–80%), geraniol (1.2%-4.6%), terpinen-4-ol (3%), α-terpineol (0.5%), *γ*-terpinene (1–8%), *r*-cymene (3.5%), limonene (0.5%-4.0%), *α*-pinene (0.2%-8.5%), camphene (1.4%), myrcene (0.2%-2.0%), camphor (0.9%-4.9%), geranyl acetate (0.1%-4.7%), linalyl acetate (0%-2.7%)	[135]
*Cymbopogon citratus*	Lemon grass	citral α (40.8%), citral β (32%), nerol (4.18%), geraniol (3.04%), citronellal (2.10%), terpinolene (1.23%), geranyl acetate (0.83%), myrecene (0.72%), terpinol (0.45%), methylheptenone (0.2%), borneol (0.1–0.4%), linalyl acetate (0.1%), *α*-pinene (0.07%), β-pinene (0.04%), limonene (traces), linalool (traces)	[140]
*Cymbopogon winterianus*	Citronella grass	Myrcene (3.3%), limonene (2.2%), (E)-β-ocimene 0.7%), allo-ocimene (0.2%), citronellal (26.5%), (E)-isocitral (0.2%), citronellol (7.3%), nerol (0.4%), neral (0.5%), geraniol (16.2%), geranial (0.7%), citronelyl acetate (2.5%), neryl acetate (0.1%), geranyl acetate (3.4%), β-elemene (4.4%), β-ylangene (0.3%), β-gurjunene (0.2%), aromadendrene (0.1%), *α*-humulene (0.1%), *cis*-cadina-1,(6),4-diene (0.1%), *cis*-muurola-4,(14),5-diene (0.1%), *γ*-muurolene (0.1%), germacrene D (1.1%), *trans*-muurola-4,(14),5-diene (0.1%), viridiflorene (0.1%), *α*-muurolene (0.4%), *γ*-cadinene(0.4%), *δ*-cadinene (2.5%), zonarene (0.1%), *α*-cadinene (0.1%), elemol (14.5%), 10-epi-*γ*-eudesmol (0.1%), 1-epi-cubenol (0.1%), *γ*-eudesmol (0.8%) epi-o-cadinol (0.5%), epi-o-muurolol (0.7%), β-eudesmol (0.2%), *α*-eudesmol (0.2%), *α*-cadinol (2.7%)	[142]
*Dysphania graveolens*	Fetid goosefoot	(Z,Z)-farnesol (16.01%), *γ*-terpineol (11.11%), cadine-4,11-dien-15-ol (8/34%), phytol (31.30%), ascaridol (32.21%), carvacrol (13.42%), carvacrol (17.45%), ascaridol (13.15%), *p*-cymene (12.79%)	[174]
*Hyptis* *mutabilis*	Tropical bushmint	*α*-thujene (1.6–6.0%), *α-*pinene (1.0–7.2%), 4(10)-thujene (2.027%), β-pinene (1.915–7.906%), 1-octen-3-ol (0.774–1.27%), limonene (1.34–1.731%), terpinene (0.199–0.715%), (+)-1-terpinen-4-ol (0.395–0.886%), *α*-copaene, (1.694–2.647%), β-bourbonene (0.787–1.532%), β-cubebene (0.693–1.172%), (-)-β-elemene (0.552–0.789%), E-caryophyllene (10.839–13.948%), *α*-caryophyllene (2.434–3.95%), germacrene D (6.936–14.968%), bicyclogermacrene (7.845–10.895%), germacrene A (0.591–0.906%), *γ*-cadinene (0.994%), cubebol (1.085–2.06%), (+)-*δ*-dadinene (1.188–2.166%), spathulenol (1.401%), germacrene D-4-ol (0.875%), (-)-globulol (11.604–26.61%), (-)-globulol (11.604–26.61%)	[144]
*Lavandula angustifolia*	English lavender	Linalool (33.3%), linalyl acetate (38.5%), caryophyllene (3.9%), myrcene (3.9%), *trans*-ocimene (2.4%), lavandulyl acetate (2.2%), terpinen-4-ol (2.1%)	[148]
		Linalyl acetate, linalool, (Z) β—ocimene, lavandulyl acetate, terpinen-4-ol	[6]
*Lippia alba*	Bushy lippia	Citral, linalool, β-caryophyllene; tagetenone; limonene, carvone, dihydrocarvone, piperitone, piperitenone; myrcene; *γ*-terpinene; camphor-1,8-cineole; estragole	[175]
*Matricaria chamomilla*	Chamomile	Isobutyl angelate, isoamyl angelate, methallyl angelate, isobutyl isobutyrate, methylpentyl angelate, *trans*-pinocarveol, pinocarvone	[6]
*Melissa officinalis, L.*	Common balm; lemon balm	Geranial (44.20%), neral (30.20%), citronellal (6.30%)	[158]
*Mentha x piperita*	Peppermint	Menthol, menthone, (+/-)-menthyl acetate, 1,8-cineole, limonene, β-pinene, β-caryophyllene	[159]
*Nigella sativa, L.*	Black cumin	Thymoquinone (63%), *p*-cymene (23%), *α*-pinene (<14%)	[169]
*Piper guineense*	Pepper	Linalool (41.8%), 3,5-dimethoxytoluene (10.9%)	[161]
*Rosmarinus officinalis*	Rosemary	*p*-Cymene (44.02%), linalool (20.5%), *γ*-terpinene (16.62%), thymol (1.81%), β-pinene (3.61%), *α*-pinene (2,83%), eucalyptol (2.64%), β-caryophyllene (0.11%)	[49]
*Salvia officinalis*	Common sage	1,8-cineole (7.45–9.69%), camphor (18.08–25.11%), linalool 0.31–0.51%), *α*-thujone (21.48–22.19%), β-thujone (8.78–17.70%), *α*-pinene (0.20–6.48%), β-pinene (1.55–3.74%), camphene (3.45–7.09%), myrcene (1.13–1.97%), limonene (1.87–1.91%), *γ*-terpinene (0.13–1.66%), terpinolene (0.34–0.87%), bormeol (1.60–2.88%), bormyl acetate (0.41–0.74%), β-caryophyllene (2.70–6.18%), *α*-humulene (2.53–4.21%), caryophyllene oxide (0.08–0.62%), viridiflorol (0.13–0.19%)	[176]
*Salvia sclarea*	Clary sage	Linalyl acetate (31.07%), linalool (23.11%), *α*-terpineol (7.03%), geranyl acetate (3.53%), sclareol (3.34%), germacrene D (2.92%), spathulenol (2.16%), caryophyllene oxide (2.01%), neryl acetate (1.87%), *trans*-β-caryophyllene (1.72%), β-myrcene (1.65%), geraniol (1.21%), nerol (1.02%), *α*-eudesmol (0.78%), limonene (0.58%), manool (0.57%), *cis*-β-ocimene (0.55%), *α*- and β-thujone (0.09%)	[177]
*Syzygium aromaticum*	Clove	Eugenol (71.56%), eugenol acetate (8.99%), caryophyllene oxide (1.67%), nootkatin (1.05%), phenol-4-(2,3-dihydro-7-methoxy-3-methyl-5-(1-propenyl)-2-benzofurane (0.98%), *p*-cymene (0.9%), guaiol (0.90%), thymol (0.87%), isolongifolanone (*trans*) (0.86%), 5-hexene-2-one (0.67%), benzene-1-butylheptyl (0.55%), hexadecanoic acid (0.50%), 9,17-octadeca-dienal (0.24%), octadecanoic acid butyl ester (0.33%), dodecatrienoic acid-3,7,11-trimethylethyl ester (0.38%), vitamin E acetate (0.43%)	[165]
*Tagetes minuta, L.*	Black mint	β-phelandrene, limonene, β-cimene, dihydrotagetone, tagetone, tagetenone	[178]
*Thymus capitatus*	Spanish oregano	*α*-thujene (1.64–7.92%), anisole (1.24%), *p*-cymene (3.71%), *trans*-β-ocimene (0.07–1.03%), *γ*-terpinene (0.76–16.18%), *α*-terpinolene (0.25–0.5%), 2-ethyl-4-methyl anisole (2.63%), thymol (0.07–1.77%), carvacrol (24.28%-58.56%), phenol,2,3,5,6-tetra-methyl (2.18–5.54%), hexanoic acid, hexyl ester (0.84%), β-caryophyllene (7.41–8.59%), aromadendrene (0.78%), *α*-humulene (1.82–5.20%), ledene/viridiflorene (1.63–6.57%), *cis*-*α* bisabolene (0.71–1.07%), *γ*-cadinene (0.69–1.93%), *δ*-cadinene (0.79%), (-)-spathulenol (1.03%), caryophyllene oxide (6.26–10.43%), iso aromadendrene epoxide (0.82–2.27%), α cadinol (0.25%), vulgarol B (1.11%)	[169]
*Valeriana officinalis, L.*	Valerian	Carene (0.29%), *α*-thujene (4.18%), 6-isopropyl-1-methyl bicycle[3,1,0]hexane (14.19%), sabinene (2.55%), *p-*cymene (0.43%), limonene (1.26%), camphor (0.19%), borneol (3.54%), L-myrtanol (0.81%), *α*-methyl 4(1′,1′-methyl ethyl) phenol (2.49%), bornyl acetate (23.93%), sabinol (1.70%), *α*-terpineol (1.20%), β-caryophyllene (0.82%), β-gurjunene (1.16%), humulene (0.40%), *trans*-caryophyllene (0.28%), nerolidol (0.78%), elemene (0.45%), bornyl isovalerianate (0.36%), azulene furan (0.58%), stereoisomer of ramie enol (1.46%), 4a,8-dimethyl-*α*-isopropyl naphthyl ketone (2.77%), tetramethyl-4-hydroxyl cyclopropane naphthalene (1.26%), ledol (1.22%), guaiol (4.73%), valerone (1.14%)	[179]

**Table 4:** Terpenes and their biological properties.

Chemical Compound	Classification	Species Tested	Application and Results	References
13-acetyl solstitialin	Sesquiterpene	Review	Anti-cancer	[180]
8*α*-Acetoxyzaluzanin C	Sesquiterpene	Review	Anti-microbial, anti-viral	
Angeloylenolin	Sesquiterpene	Review	Anti-proliferative	
Arglabin	Sesquiterpene	Review	Anti-cancer, anti-malarial	
Arguerin B	Sesquiterpene	Review	Anti-cancer	
Artemisin	Sesquiterpene	Review	Anti-cancer, anti-malarial	
*α*-asarone		Rats	Anxiolytic like effect; alleviated epilepsy; anti-depressant	[92,99,101]
β-asarone			Anti-depressant	[100,101]
(-)-Borneol	Monoterpene	Review	Anti-bacterial	[181]
(+)-Borneol	Monoterpene	Review	Anti-bacterial	[181]
Camphor	Monoterpene	Rats	Anti-bacterial	[181]
Carotenoids	Tetraterpene	Review	Anti-oxidant, photoprotectant	[182]
Carvacrol	Monoterpene	Mice	Anxiolytic like effect; anti-microbial, pro- and anti-apoptotic	[92,180]
L-Carveol	Monoterpene	Review	Anti-bacterial; anti-oxidant	[104,181]
L-Carvone	Monoterpene	Review	Anti-bacterial	[181]
β–Caryophyllene	Sesquiterpene	Mouse	Improved re-epithelialization of cutaneous wound, stimulated cell proliferation and migration; Anti-inflammatory, analgesic; ligand of CB2; Anti-carcinogenic; Anxiolytic-like effect; allelopathic	[2,5,92,180]
Centaurepensin A	Sesquiterpene	Review	Anti-microbial, anti-viral	[180]
Chlorocyanerin	Sesquiterpene	Review	Anti-microbial, anti-viral	[180]
1,4-Cineole	Oxides	Review	Anxiolytic-like effect	[92]
1,8-Cineole	Oxides	Humans; rat	Improved cognitive function; increased skin flap survival	[52,183]
Citral	Monoterpene	Mouse, silver catfish	Enhanced microbiota, especially of Lactobacillus, in the gut; anxiolytic-like effect; anti-microbial, anti-inflammatory, anti-cancer; anti-bacterial; anesthetic	[71,92,152,153,180,181]
Citronellal	Monoterpene	Mouse	Stimulated glucagon-like peptide 1 secretion in mouse intestinal tissues and in cultured enteroendocrine cells; anti-bacterial	[62,63,64,65,66,67,68,69,70,71,72,73,74,75,76,77,78,79,80,81,82,83,84,85,86,87,88,89,90,91,92,93,94,95,96,97,98,99,100,101,102,103,104,105,106,107,108,109,110,111,112,113,114,115,116,117,118,119,120,121,122,123,124,125,126,127,128,129,130,131,132,133,134,135,136,137,138,139,140,141,142,143,144,145,146,147,148,149,150,151,152,153,154,155,156,157,158,159,160,161,162,163,164,165,166,167,168,169,170,171,172,173,174,175,176,177,178,179,180,181]
Cnicin	Sesquiterpene	Review	Anti-microbial	[180]
Coronopilin	Sesquiterpene	Humans	Anti-cancer	[180]
Cyclohexyl salicylate			Stimulated cytokinesis, cell migration, regeneration of keratinocytes, phosphorylation of AKT and Chk-2, and secretion of IL-1; ligand of OR2A4/7	[67]
*m*-Cymene	Monoterpene	Review	Anti-bacterial	[181]
*p*-Cymene	Monoterpene	Review	Analgesic, anti-inflammatory, anti-nociceptive	[184]
Cynaropicrin	Sesquiterpene	Review	Anti-cancer	[180]
Eugenol	Monoterpene	Review	Anti-bacterial	[181]
Eupalinin	Sesquiterpene	Review	Anti-cancer	[180]
β-*cis*-farnesene	Sesquiterpene	Review	Allelopathic	[180]
Geraniol	Monoterpene	Mouse	Stimulated glucagon-like peptide 1 secretion in mouse intestinal tissues and in cultured enteroendocrine cells; Strong analgesic impact as morphine, measured by the latency to show writhing after intraperitoneal injection of acetic acid; IP injection; anti-depressant impact, measured by the immobility time in forced swimming tests and tail suspension tests, lower corticosterone level; gavage; anti-tumor, anti-microbial, anti-oxidant, anti-inflammatory	[62,77,78,79,180,181]
Helenalin	Sesquiterpene	Review	Anti-cancer	[180]
Humulene	Sesquiterpene		Suppressd hepatocellular carcinoma cell proliferation	[185]
β –ionone		Humans	Melanin synthesis; ligand of OR51E2	[66]
Isomontanolide	Sesquiterpene	Review	Anti-microbial, anti-biofilm	[180]
Isononyl alcohol		Humans	Stimulated cell migration, phosphorylation of Hsp27, AMPK1, and p38MAPK, and secretion of IL-6; ligand of OR51B5	[67]
Linalool	Monoterpene	Mouse Silver catfish	Anxyolitic effect; inhalation enhanced the microbiota, especially of Lactobacillus, in the gut; anti-bacterial; sedative, anesthetic effect	[3,71,151,152,153,181]
Limonene	Monoterpene	Mouse; review	Enhanced the microbiota, especially of *Lactobacillus*, in the gut, Ligand of Olfr56 (human homolog, OR2V1); anti-depressant, anti-nociceptive, anti-diabetic, anti-ulcerogenic	[71,180]
Menthol	Monoterpene	Review	Anti-microbial, radioprotective, anti-oxidant, analgesic;	[180]
Montanolide	Sesquiterpene	Review	Anti-microbial, anti-biofilm	[180]
Myrcene	Monoterpene	Mouse	Anxiolytic-like effect; analgesic, anti-inflammatory, anti-oxidant, anti-bacterial	[92,180]
Ocimene	Monoterpene	Review	Wound healing, anti-inflammatory, abolished or reduced edema, hyperemia, laceration, hemorrhage; anti-microbial	[180,181]
Parthenolide	Sesquiterpene	Review	Anti-inflammatory	[180]
Perillyl alcohol		In vitro	Anti-oxidant	[104]
2-Phenylethanol		Mouse	Decreased immobility time in tail suspension test	[48]
*α*–Pinene	Monoterpene	Rat; review	Increased survival of skin flap; anxiolytic-like effect; anti-microbial	[92,180,181,183]
β–Pinene	Monoterpene	Review	Anti-microbial	[181]
Sandalore	Synthetic sandalwood odorant	Humans	Stimulated chemotaxis in human keratinocyte cells; ligand of OR2AT4; Suppressed apoptosis and enhanced longevity of hair; ligand of OR2AT4	[59,63]
Terpineol	Monoterpene	Review	Anti-bacterial	[181]
Tanachin	Sesquiterpene	Review	Anti-bacterial	[180]
Tavulin	Sesquiterpene	Review	Anti-bacterial	[180]
Tourneforine	Sesquiterpene	Review	Cytotoxic	[180]
Thymol	Monoterpene	Review	Anti-fungal, anti-parasitic, anti-septic; anti-bacterial	[180,181]

## Abbreviations

CAP	Compound action potentials
CB2	Cannabinoid receptor 2
GABA	Gamma aminobutyric acid
TRP	Transient receptor potential channels
TRPA	Transient receptor potential channels subfamily A
TRPM	Transient receptor potential channel metastatin
TRPV	Transient receptor potential channel vanilloid
MAPK	Mitogen-activated protein kinase
AKT	Protein kinase B
Chk-2	Checkpoint kinase 2
IL-1	Interleukin 1
IL-6	Interleukin 6
Hsp27	Heat shock protein 27
AMPK1	Adenosine Monophosphate-activated protein kinase 1
P38MAPK	protein kinases and the 38-kDa mitogen-activated protein kinase
Wnt	Wingless related integration site
Gli1	Glioma associated oncogene
Adamts	Disintegrin and metalloproteinase with thrombospondin motifs
Bambi	bone morphogenetic protein and activin membrane-bound inhibitor homolog
Msx2	Msh homeobox2
Dlx3	Distalless 3
Padi1	peptidyl arginine deiminase 1
Hoxc13	Homeobox C13
S100a	a family of calcium-binding cytosolic proteins
BrdU	Bromodeoxyuridine
Lgr5	Leucine-rich repeat-containing G-protein coupled receptor 5
IP	Intra-peritoneal
CNS	Central nervous system
5-HT	5-hydroxytryptamine
TBPS	Tert-butylbicyclophosphorothionate; GABA receptor antagonist
EEG	electroencephalography
AOB	Accessory olfactory bulb
MOB	Main olfactory bulb
NG	Necklace glomeruli
OCAM	Olfactory cell adhesion molecule
GC-D	Receptor guanylyl cyclase
TAAR	Trace amine-associated receptors
TMT	Trimethyl-thiazoline
SBT	2-sec-tutyl-dihydrothiazole
DHB	dehydro-exo-brevicomine
